# Analysis of Factors Influencing Corrective Power of Akin’s Osteotomy in 2D Plain Radiographs: What to Consider to Obtain Good Correction in Hallux Valgus Surgery

**DOI:** 10.3390/diagnostics15131618

**Published:** 2025-06-26

**Authors:** Enrique Adrian Testa, Alberto Ruiz Nasarre, Fernando Alvarez Goenaga, Daniel Poggio Cano, Annamaria Porreca, Albert Baduell, Ruben Garcia Elvira, Miki Dalmau-Pastor, Pablo Ruiz Riquelme

**Affiliations:** 1 Orthopaedic Surgery and Traumatology, Ospedale Regionale di Bellinzona e Valli, EOC, 6500 Bellinzona, Switzerland; 2 Faculty of Biomedical Sciences, Università della Svizzera Italiana, USI, 6900 Lugano, Switzerland; 3 Ars Medica Clinic, 6929 Gravesano, Switzerland; 4 Human Anatomy and Embryology Unit, Department of Pathology and Experimental Therapeutics, School of Medicine and Health Sciences, University of Barcelona, 08007 Barcelona, Spain; mikeldalmau@gmail.com; 5 Foot and Ankle Unit, Department of Orthopedic and Traumatology, Hospital Sant Rafael, 08035 Barcelona, Spain; aruizn.hsrafael@hospitalarias.es (A.R.N.); alvarez173@gmail.com (F.A.G.); 6 Foot and Ankle Unit, Department of Orthopedic and Traumatology, Hospital Clinic, University of Barcelona, 08007 Barcelona, Spain; dpoggio@clinic.cat (D.P.C.); baduell@clinic.cat (A.B.); rgarciae@clinic.cat (R.G.E.); 7 Department of Human Sciences and Promotion of the Quality of Life, San Raffaele, 00166 Rome, Italy; annamaria.porreca@uniroma5.it; 8 Unit of Clinical and Molecular Epidemiology, IRCCS San Raffaele Roma, 00163 Rome, Italy; 9 MIFAS by GRECMIP (Minimally Invasive Foot and Ankle Society), 33700 Merignac, France; 10 Department of Orthopedic and Traumatology, Las Condes Clinic, Santiago 7591047, Chile; pabloruizr@gmail.com; 11 School of Medicine, Finis Terrae University, Santiago 7501015, Chile

**Keywords:** Akin osteotomy, proximal phalangeal osteotomy, hallux valgus, valgus interphalangeus

## Abstract

**Background/Objectives:** Akin osteotomy, in the context of corrective surgery for hallux valgus, is an effective tool available to surgeons. However, few studies have thoroughly investigated the anatomical and technical characteristics to be considered in order to perform an optimal osteotomy. This cross-sectional observational study aims to identify the ideal site for performing Akin osteotomy and to identify the factors that influence its corrective power. **Methods:** To this end, an analysis was conducted on a random sample of 100 patients (186 feet) who underwent X-rays without surgical treatment. Variations in the width between the metaphysis and diaphysis were measured at five different points. For each cut level, corresponding to wedge bases of 2, 3 and 4 mm, three corrective angles were calculated. In addition, the distance between the cut line and the joint was recorded. **Results:** The base width ranged from 12.6 to 23.2 mm, showing greater variability in the metaphyseal region. The corrective power of the osteotomy showed wide variability, ranging from 5.9 to 18.4 degrees. Four determining factors emerged: the width of the base, the inclination of the medial cortex, the height at which the cut is made and the thickness of the wedge of bone removed. The data obtained suggest that osteotomy should not be performed less than 10 mm from the joint line to avoid the risk of joint invasion. **Conclusions:** In conclusion, there is no universally ideal site for performing an Akin osteotomy: the choice depends on the degree of correction desired, which in turn is influenced by the factors identified in the study.

## 1. Introduction

The Akin procedure is a surgical technique aimed at correcting the axis of the proximal phalanx of the big toe. It is based on a wedge-shaped medial osteotomy, with the aim of correcting the interphalangeal angle and reducing the tension exerted by the tendons of the long extensor and flexor muscles of the big toe, which contribute to the maintenance and progression of the deformity [1,2,3,4]. This procedure is performed frequently in clinical practice and is often associated with osteotomies of the first metatarsal, especially in surgical procedures for hallux valgus (HV), where it plays a complementary role in improving the overall alignment of the first ray and achieving a more stable and lasting result.

Despite the progressive increase in publications dedicated to hallux valgus and its surgical management, the scientific literature still lacks studies that analyze in detail the specific technical and biomechanical characteristics of the Akin procedure. In particular, there is a poor understanding of the anatomical and radiological parameters that may influence its corrective power, as well as the intrinsic limitations of the technique in its various modifications [2,3,4,5,6,7,8].

A first attempt to analytically quantify the effect of the correction was made in the early 1990s by Frey et al., who proposed a mathematical model to estimate the angular variation obtainable through wedge osteotomies with base widths of 3, 5 and 8 mm, taking into account the total length of the proximal and distal phalanges [7]. However, Frey’s approach is limited to a theoretical assessment related to the geometry of the osteotomy and does not explore in depth what other factors may contribute to the actual clinical and radiological outcome. Since the publication of this study, no new research has emerged that systematically examines the influence of these factors on the outcome of Akin osteotomy.

In light of this gap, we believe it is essential to explore whether there are additional measurable elements that may influence the effectiveness of the correction achievable with the Akin procedure. In particular, it is necessary to develop an analysis based on tools commonly used in orthopedic practice, such as two-dimensional radiographs, which are the most accessible and widespread diagnostic modality in preoperative planning and postoperative follow-up. The aim of this study is therefore twofold: on the one hand, to identify the optimal site for performing the Akin osteotomy in order to maximize its corrective power; on the other hand, to determine more precisely which radiological and anatomical parameters significantly influence the extent of the correction obtained, in order to provide the orthopedic surgeon with practical, evidence-based guidance for more effective surgical planning.

## 2. Material and Methods

This is a retrospective cross-sectional study of 100 patients (186 feet) scheduled for HV correction surgery in a single center between 2011 and 2018. We follow the Strobe guidelines for cross-sectional studies (https://www.equator-network.org/reporting-guidelines/strobe/) accessed on 23 January 2022. All cases were randomly selected using the Randomizer program (https://www.randomizer.org/) from a data set of 523 procedures performed for HV correction. Exclusion criteria were previous foot surgery or fracture at the level of the first ray, presence of hallux rigidus stage II or higher (according to Coughlin–Shurnas classification) or other deforming pathologies of the proximal phalanx, presence of bone or soft tissue tumor pathologies at phalangeal or metatarso-phalangeal level, absence of weight-bearing radiographs, patients under the age of 18 [9].

**Figure 1:**
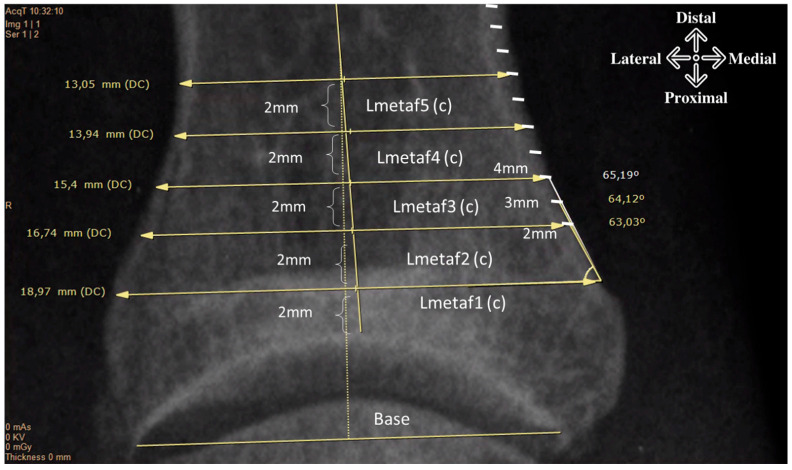
The image shows the subdivision of the proximal phalanx of a link foot into five levels. Starting 2 mm from the articular vault, the width of the base (c) is measured by drawing a line parallel to the articular line (base). For each level, the medial inclination angle is measured at 2, 3 and 4 mm (white marks).

We obtained generic demographic data (age, sex and side), and X-ray measurements were taken to obtain the angulation of the phalanx with respect to the articular line using the distal articular set angle (DASA) and the interphalangeal oblique angle (IPOA). We then calculated the width of the phalanx from the proximal metaphysis to the diaphysis at five different points: two millimeters from the articular surface at the higher level and then at 4, 6, 8 and 10 mm moving distally (Figure 1).

**Figure 2:**
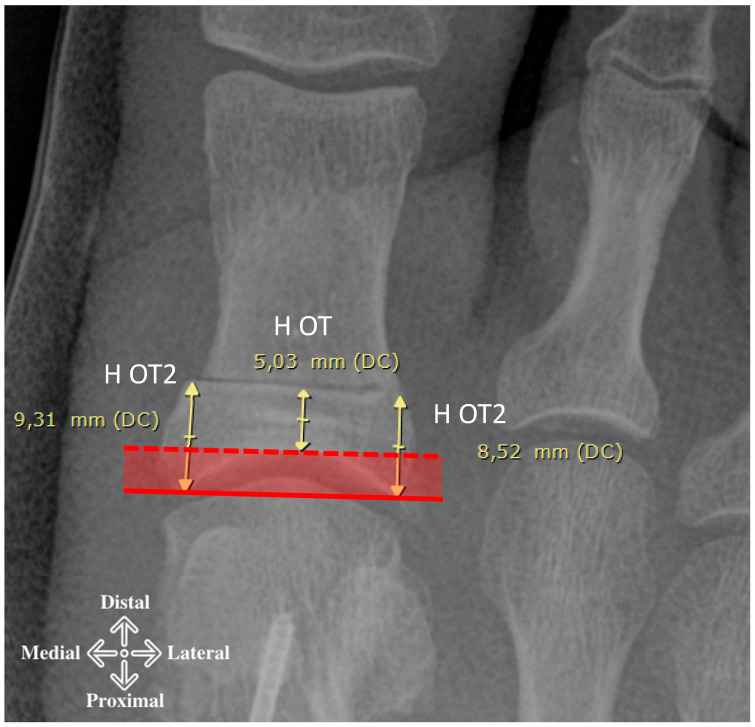
Distance of the Akin osteotomy from the joint and vault. The height of the osteotomy level is measured in relation to the articular apex H OT (dashed line) and the articular line H OT2 (solid line). H OT2 is calculated from the average of the two distances between the articular line and the medial and lateral end of the cut.

We also measured the distance between the proximal osteotomy cut and the joint at the more proximal (H OT2) and more distal (H OT) level (joint line and apex of the joint dome on the phalanx) (Figure 2). By subtracting these two parameters, we obtained the maximum articular depth of the joint.

**Figure 3:**
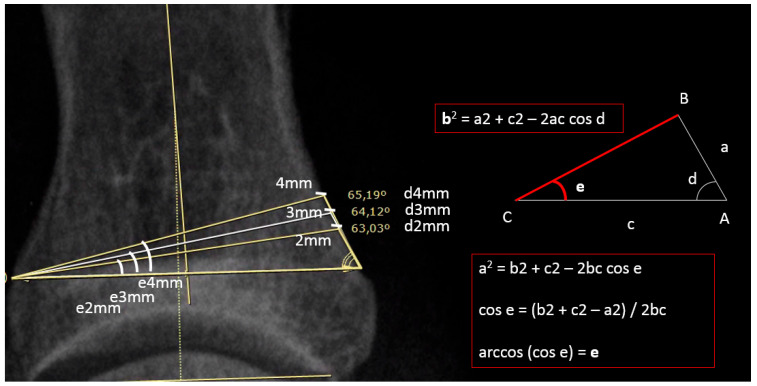
Schematic representation of Carnot’s theorem and its application. The image shows the trigonometric correlation between side lengths and angle widths according to Carnot’s theorem. In the cut level in the example, 3 different base thickness amplitudes (2, 3 and 4 mm) correspond to 3 different medial cortical tilt angles (d) and 3 different subtractive correction angles (e).

We have considered the subtractive wedge in the dorso-plantar plane. In such a plane, the wedge behaves like a triangle (Figure 3), the base of which is possible to measure the length of (c), corresponding to the width of the meta/diaphysis, the length of the short side of which corresponds to the choice of the wedge thickness (in our case 2 mm, 3 mm and 4 mm), and the angle subtended between these two sides corresponds to the medial cortical inclination angle (d). Therefore, 3 angles were measured for each cut level. We calculated the median of the angles at the different cut levels, obtaining for each phalanx 15 angles (3 for each of the 5 levels).

Through a series of mathematical variables derived from Carnot’s cosine theorem (Figure 3), it is possible to obtain the subtractive correction angle (e).

### 2.1. Statistical Report

Descriptive statistics are reported as median and 1st and 3rd quartiles or by mean, standard deviation, minimum value and maximum value following the variable’s normal distribution verified by Shapiro–Wilk test. The Mann–Whitney U-test was applied to investigate whether there were significant differences between gender for base widths (c) of the phalanx from proximal (Lbase) to distal (LMetaf5). Spearman’s rho correlation coefficient, with listwise deletion, was calculated to compare the relationship of the continuous parameters. The significance level was set at α = 0.05. All statistical analyses were performed by R statistical environment version 3.6.

### 2.2. Ethics Statement

Permission to perform this study was obtained from the Internal Review Board (Ethics Committee PR-2022-3). This article does not contain any experimental studies with human participants or animals performed by any of the authors.

## 3. Results

We analyzed the radiographs of 186 feet (166 female and 20 male; 95 left and 91 right feet) in 100 patients (90 female and 10 male; mean age 60.1 (25–83) years). From the images, we observed that the proximal cut lines were performed, on average, 8.93 (5.38–12.85) mm from the articular line and, on average, 5.29 (1.57–9.20) mm from the articular dome (Figure 2). The mean of the depth joint at the base of the phalanx was 3.71 (2.27–7.85) mm.

Table 1 shows the mean values of the widths of the base of the proximal phalanx and the metaphyseal–diaphyseal region distal to the joint dome by 2 mm and parallel to the joint line (Figure 1). It is observed that the width of the base varies between 12.6 and 23.2 mm, but when looking at the metaphyseal region (L1-L2-L3), there is greater variability of the means in the metaphyseal region (15.15–18.5 mm) and less in the diaphyseal region (12.16–13.26 mm). Table 2 shows that for all levels analyzed, although there is an imbalance in the sample number (166 f: 20 m), there is a statistically significant difference in the width of the metaphysis/diaphysis between males and females.

Table 1 shows the mean medial cortical angle (d) measured at 2, 3 and 4 mm, in relation to the level of the supposed osteotomy cut (Figure 1) and the mean correction angles (e) calculated at different levels and with different shear amplitudes.

We can observe that for 2 mm of wedge, the median corrective power (e) varies from 5.96 (4.40–7.38) degrees at the most proximal level (2 mm from the articular dome), progressively increasing up to 9.38 (7.06–12.84) degrees at the diaphyseal level (10mm from the dome). Similarly, for 3 mm of wedge, the median correction angle (e) varies from 9.0 (6.82–11.29) degrees up to 13.9 (10.77–19.02) degrees and for 4 mm of wedge, from 12.1 (9.38–15.3) degrees up to 18.4 (14.12–24.5) degrees.

Table 3 shows the correlations between the DASA, IPOA, and cortical tilt angle (d) and the calculated correction angle (e) for Akin’s osteotomy. The indicators show that there is a strong correlation between the amplitude of the medial cortical angle (d) and the amplitude of the DASA and IPOA, especially from the second level (4 mm from the joint vault). In contrast, such a strong correlation is not observed for the calculated correction angles (e).

## 4. Discussion

We have achieved our goal of identifying the main factors that influence the corrective power of Akin osteotomy and, consequently, of defining the areas where it is most advisable to perform it. The main conclusion we can draw from this study is that certain elements—which, to our knowledge, have not been highlighted in other studies—have a significant influence on the corrective capacity of osteotomy. In particular, the width of the phalanx base, the inclination of the medial cortex and the level at which the cut is made are crucial aspects to consider during preoperative planning. The metaphysical region has a complex morphology, sometimes resembling a tilted wine glass, while the diaphyseal zone tends to ‘normalize’, becoming thinner and taking on a more cylindrical appearance [4,9,10,11,12]. Previous studies have shown that the average width of the phalanx base can vary between 13.6 mm and 15.2 mm at distances from the joint line between 17.8 mm and 19.8 mm, with slightly lower values in females and no significant differences between different ethnic groups [11,12]. The inclination of the medial cortex shows marked variability along the phalanx: it tends to be more inclined proximally, due to the metaphyseal curvature, than in the diaphyseal area, where it is more regular. It follows that to achieve the same degree of correction, an osteotomy performed proximally requires a wider wedge base than one performed distally.

In analogy with studies performed on long bones, it is confirmed that the effectiveness of osteotomy—expressed as the angular correction axis (ACA)—increases as the cut approaches the center of rotation of the angle (CORA) [13,14]. Applying this concept to the proximal phalanx of the hallux, more distal osteotomies seem to guarantee greater corrective power than more proximal ones, an observation confirmed by our data.

Hence, carefully examining preoperative radiographs, assessing the shape of the phalangeal hemibase, any morphological alterations (such as hypo- or hyperplasia) and the relationship between the phalangeal axis and the joint line is important. Our data show a correlation between the variation in the DASA and IPOA and that of the medial cortical angle; however, these variations do not correlate strongly with the desired angle of correction (Table 3). This suggests that the choice of wedge size should not be based solely on the DASA and IPOA.

With regard to the level of the osteotomy cut, the literature is unclear. Several authors recommend performing the cut 5–7 mm from the joint to avoid violating the joint dome. However, it is not always specified whether this distance refers to the joint line or the apex of the dome [2,4,7,10,15]. Our study shows that the average depth of the joint dome is approximately 3.71 mm (range: 2.2–7.85 mm). To ensure a safety margin of at least 2 mm, we suggest positioning the cutting line no less than 10 mm from the joint line. This distance may need to be increased in the presence of bulky fixation systems, such as staples or large-headed screws.

One of the shortcomings of the literature on Akin osteotomy is the absence of a mathematical model that accurately describes the relationship between the wedge thickness and degree of correction. The study by Frey et al., based on 45 cases, suggests that a 3 mm wedge produces a correction of 8°, a 5 mm wedge leads to 16° and an 8 mm wedge leads to 24°. However, neither the length of the phalanges nor the bone morphology on which the calculations were performed is specified. Furthermore, the authors base their findings on osteotomies performed 5–7 mm from the joint [7].

Our data only partially confirm these estimates. For example, with 3 mm wedges, we obtained corrections ranging from 6.7° to 19.0°, showing much greater variability than the 8° indicated by Frey. This variability is even more evident with 2 mm wedges (4.4–12.8°) and 4 mm wedges (9.4–24.5°). This highlights the significant influence of phalangeal morphology on the correction that can be achieved, an aspect that previous studies do not seem to have adequately considered [4,7,10,12].

### Limitations

The interpretation of the data must take into account a number of assumptions in order to make the calculations feasible: firstly, that the cut line is parallel to the joint line; this choice implies that if it is decided to make oblique cuts, different values will be obtained from those presented in this study. Secondly, the rotation of the phalanx must be considered in radiographic biplanar examinations. It should be emphasized that the Akin osteotomy is normally performed after the metatarsal osteotomy, which can in turn create a variation in the rotation of the phalanx with respect to the preoperative planning. Therefore, we believe that in the case of excessive alterations (normally in large deformities), this effect should not be underestimated.

**Figure 4:**
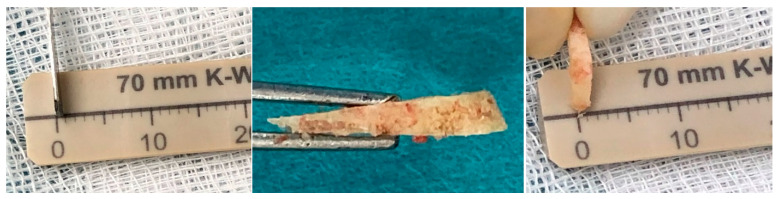
The image shows how the thickness of the saw is less than 1 mm and how the subtracted wedge appears after removal. In the case presented, a 2 mm osteotomy was performed but the subtracted wedge is slightly smaller in size. This is due to the thickness of the saw and its oscillation.

Finally, we have chosen 2 mm as the minimum value for the length of the base of the osteotomy triangle because the thickness of the saw and its oscillation, even without removing any bone segments, leads to a minimum approximate opening of 2 mm of the medial cortex (Figure 4). The maximum value chosen was 4 mm, as we rarely perform subtractions above this value. However, an approximate calculation can be made for higher values, according to our model, using Carnot’s theorem.

## 5. Conclusions

**Figure 5:**
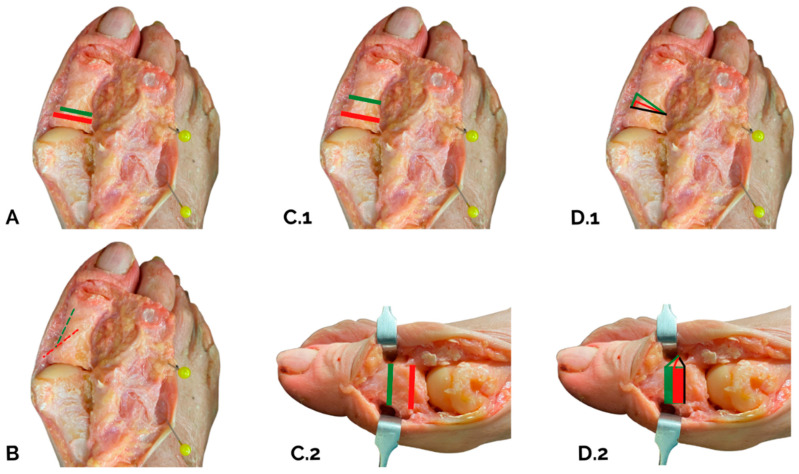
Factors that more (green) or less (red) significantly influence the magnitude of Akin’s osteotomy and thus the power of correction are illustrated. The following factors are highlighted in these cadaveric preparations: (**A**) Width of the base of the first phalanx. (**B**) Tilt of the medial cortex. (**C.1**,**C.2**) Position of the osteotomy (proximal to distal). (**D.1**,**D.2**) Width of the base of the subtraction osteotomy.

1. The presented study provides an approximation of the corrective power of Akin’s osteotomy. This power depends on several factors including the width of the base, the inclination of the medial cortical, the height at which the osteotomy is performed and the width of the base of the subtraction wedge (Figure 5).
2. The data suggest not performing the osteotomy within 10 mm of the joint line to avoid intra-articular extension.
3. Greater angle correction is achieved if the cut is more diaphyseal versus metaphyseal and if the subtraction wedge inclination has a less acute versus more acute angle.
4. The DASA and IPOA calculated on 2D plain radiographs would appear to be unreliable factors for planning purposes.
5. Finally, the greater the width of the subtraction wedge, the greater the correction. It is important to consider all factors together, and this explains why different magnitudes of correction can be achieved, as observed in this study.

Corrections can be obtained, as follows:

- Between 5.9 and 9.3 degrees with 2 mm base wedges.
- Between 9.0 and 14 degrees with 3 mm wedges.
- Between 12.1 and 18.4 degrees with 4 mm wedges, depending on the starting position of the osteotomy (metaphyseal or diaphyseal area) and the inclination of the medial cortical wall.

## Figures and Tables

**Table 1:** Descriptive statistics for the variables in the study presented as mean, standard deviation (SD), minimum and maximum value.

Variables (N = 186)	Mean	SD	Min	Max	
DASA, °	1.11	3.96	−12.00	12.00	Measured angles
IPOA, °	8.66	5.00	−4.00	25.00	
HOT, mm	5.14	1.45	1.57	8.08	Osteotomy Height
H2OT, mm	8.91	1.50	5.38	12.85	
Lbase, mm	16.71	1.48	12.66	23.23	Width of the base (c)
LMetaf1, (c), mm	18.75	1.45	15.13	23.91	
LMetaf2, (c), mm	17.43	1.62	13.57	23.95	
LMetaf3, (c), mm	15.22	1.69	11.59	21.43	
LMetaf4, (c), mm	13.41	1.56	9.98	17.96	
LMetaf5, (c), mm	12.27	1.43	8.99	15.96	
M2mm-L1, (d), °	71.54	9.68	49.98	94.06	Cortical angle measured (relative to the joint line) using short side (shear width over cortical) and increasing from 2 to 3 and 4 mm (d)
M3mm-L1, (d), °	69.46	7.65	48.29	87.57	
M4mm-L1, (d), °	67.38	6.89	44.28	87.06	
M2mm-L2, (d), °	65.36	8.16	40.95	91.07	
M3mm-L2, (d), °	66.31	7.50	43.26	90.92	
M4mm-L2, (d), °	67.26	7.67	45.57	90.76	
M2mm-L3, (d), °	71.41	8.68	51.83	92.94	
M3mm-L3, (d), °	72.93	8.20	56.25	95.53	
M4mm-L3, (d), °	74.45	8.08	57.65	98.11	
M2mm-L4, (d), °	78.66	7.98	56.89	105.57	
M3mm-L4, (d), °	80.07	7.56	63.35	103.69	
M4mm-L4, (d), °	81.47	7.37	64.08	101.81	
M2mm-L5, (d), °	84.40	7.26	67.36	102.18	
M3mm-L5, (d), °	85.49	6.90	69.82	102.66	
M4mm-L5, (d), °	86.59	6.82	70.68	103.74	
e2mm-L1, (e), °	5.92	0.53	4.40	7.38	Akin’s correction angle (with respect to joint line and cortical tilt) (e)
e3mm-L1, (e), °	8.99	0.77	6.82	11.29	
e4mm-L1, (e), °	12.09	1.02	9.38	15.30	
e2mm-L2, (e), °	6.24	0.71	4.12	8.47	
e3mm-L2, (e), °	9.61	1.05	6.70	12.73	
e4mm-L2, (e), °	13.06	1.38	9.60	16.86	
e2mm-L3, (e), °	7.41	0.93	5.07	9.94	
e3mm-L3, (e), °	11.31	1.31	7.99	14.85	
e4mm-L3, (e), °	15.24	1.65	10.76	19.49	
e2mm-L4, (e), °	8.59	1.01	5.93	11.55	
e3mm-L4, (e), °	12.95	1.44	9.20	17.28	
e4mm-L4, (e), °	17.20	1.82	12.60	22.73	
e2mm-L5, (e), °	9.40	1.05	7.06	12.84	
e3mm-L5, (e), °	14.01	1.50	10.77	19.02	
e4mm-L5, (e), °	18.42	1.90	14.12	24.49	

Akin’s correction angle (with respect to joint line and cortical tilt) (e). DASA = distal articular set angle, IPOA = interphalangeal joint obliquity angle, HOT = osteotomy height from vault, H2OT = osteotomy height from joint, (c) Lbase/LMetaf = phalanx width measured at different points, (d) M2/4mm-L1/5 = medial cortical tilt angle, (e) e2/4mm-L1/5 = Akin’s osteotomy correction angle.

**Table 2:** Mean [q1 = first quartile, q3 = third quartile] for LMetaf by gender and *p*-value from Mann–Whitney U-test.

Variables	Female	Male	*p*-Value
	N = 166	N = 20	
DASA, °	1.00 [0.00;3.00]	1.00 [1.00;3.25]	0.272
IPOA, °	9.00 [6.00;12.0]	6.50 [5.00;11.0]	0.277
Lbase, mm	16.4 [15.7;17.4]	17.0 [16.8;17.7]	0.002
LMetaf1 (c), mm	18.4 [17.7;19.4]	20.4 [18.9;21.9]	<0.001
LMetaf2 (c), mm	17.2 [16.3;18.1]	19.5 [17.7;20.7]	<0.001
LMetaf3 (c), mm	15.0 [14.0;15.8]	16.7 [15.3;19.1]	<0.001
LMetaf4 (c), mm	13.2 [12.3;14.1]	15.1 [13.7;16.9]	<0.001
LMetaf5 (c), mm	12.1 [11.2;12.9]	13.9 [12.5;15.2]	<0.001

DASA = distal articular set angle, IPOA = interphalangeal joint obliquity angle, Lbase/LMetaf = phalanx width measured at different points.

**Table 3:** Spearman’s rank correlation coefficients for DASA, IPOA and cortical tilt angle (d) and calculated correction angle (e) for Akin’s osteotomy. Computed correlation used Pearson’s method with listwise deletion. Significance code: *** = *p* < 0.001, ** = *p* < 0.01, * = *p* < 0.05.

	DASA	IPOA		DASA	IPOA
IPOA	0.458 ***		IPOA	0.458 ***	
e2mm-L1 (e)	−0.123	−0.011	M2mm-L1 (d)	−0.016	0.036
e3mm-L1 (e)	−0.159 *	−0.028	M3mm-L1 (d)	−0.112	−0.031
e4mm-L1 (e)	−0.189 **	−0.048	M4mm-L1 (d)	−0.226 **	−0.120
e2mm-L2 (e)	−0.252 ***	−0.197 **	M2mm-L2 (d)	−0.326 ***	−0.262 ***
e3mm-L2 (e)	−0.244 ***	−0.177 *	M3mm-L2 (d)	−0.426 ***	−0.302 ***
e4mm-L2 (e)	−0.218 **	−0.149 *	M4mm-L2 (d)	−0.487 ***	−0.312 ***
e2mm-L3 (e)	−0.244 ***	−0.122	M2mm-L3 (d)	−0.521 ***	−0.286 ***
e3mm-L3 (e)	−0.195 **	−0.102	M3mm-L3 (d)	−0.563 ***	−0.305 ***
e4mm-L3 (e)	−0.134	−0.075	M4mm-L3 (d)	−0.581 ***	−0.312 ***
e2mm-L4 (e)	−0.176 *	−0.062	M2mm-L4 (d)	−0.581 ***	−0.283 ***
e3mm-L4 (e)	−0.115	−0.034	M3mm-L4 (d)	−0.596 ***	−0.293 ***
e4mm-L4 (e)	−0.053	−0.003	M4mm-L4 (d)	−0.593 ***	−0.294 ***
e2mm-L5 (e)	−0.145 *	−0.022	M2mm-L5 (d)	−0.566 ***	−0.265 ***
e3mm-L5 (e)	−0.083	0.006	M3mm-L5 (d)	−0.588 ***	−0.272 ***
e4mm-L5 (e)	−0.020	0.034	M4mm-L5 (d)	−0.589 ***	−0.269 ***

DASA = distal articular set angle, IPOA = interphalangeal joint obliquity angle, M2/4mm-L1/5 = medial cortical tilt angle, e2/4mm-L1/5 = Akin’s osteotomy correction angle.

## Data Availability

The original contributions presented in the study are included in the article, further inquiries can be directed to the corresponding author.
